# 167 Community Engagement Forum: Sharing best practices in community-engaged research – ERRATUM

**DOI:** 10.1017/cts.2023.564

**Published:** 2023-06-14

**Authors:** Kaylee Rivera Gordon, Montelle Tamez, Mary Fisher, Donald E. Nease

The above abstract [1] published with Montelle Tamez’s name spelled incorrectly.

The original abstract has been corrected online to rectify this error.
